# It Takes Two: Interpersonal Neural Synchrony Is Increased after Musical Interaction

**DOI:** 10.3390/brainsci12030409

**Published:** 2022-03-20

**Authors:** Alexander Khalil, Gabriella Musacchia, John Rehner Iversen

**Affiliations:** 1 Department of Music, University College Cork, T23 X6Y0 Cork, Ireland; 2 Institute for Neural Computation, University of California, San Diego, CA 92093, USA; 3 Department of Speech Pathology and Audiology, University of the Pacific, San Francisco, CA 94103, USA; gmusacchia@pacific.edu; 4 Swartz Center for Computational Neuroscience, University of California, San Diego, CA 92093, USA; jiversen@ucsd.edu

**Keywords:** interpersonal neural synchrony, interpersonal synchrony, music, neuroplasticity, electroencephalography (EEG), hyperscanning, entrainment, interbrain phase coherence

## Abstract

Music’s deeply interpersonal nature suggests that music-derived neuroplasticity relates to interpersonal temporal dynamics, or synchrony. Interpersonal neural synchrony (INS) has been found to correlate with increased behavioral synchrony during social interactions and may represent mechanisms that support them. As social interactions often do not have clearly delineated boundaries, and many start and stop intermittently, we hypothesize that a neural signature of INS may be detectable following an interaction. The present study aimed to investigate this hypothesis using a pre-post paradigm, measuring interbrain phase coherence before and after a cooperative dyadic musical interaction. Ten dyads underwent synchronous electroencephalographic (EEG) recording during silent, non-interactive periods before and after a musical interaction in the form of a cooperative tapping game. Significant post-interaction increases in delta band INS were found in the post-condition and were positively correlated with the duration of the preceding interaction. These findings suggest a mechanism by which social interaction may be efficiently continued after interruption and hold the potential for measuring neuroplastic adaption in longitudinal studies. These findings also support the idea that INS during social interaction represents active mechanisms for maintaining synchrony rather than mere parallel processing of stimuli and motor activity.

## 1. Introduction

All interpersonal interaction requires coordination of perception and action. This coordination is observable as synchrony both at the behavioral and neural level between individuals in interaction. Synchrony of gesture, speech, breath, gaze, and gait have been found to occur spontaneously during social interaction [1,2,3] with a variety of positive effects [4]. Increased interpersonal synchrony during interaction is associated with improved communication [3,5], improved self-esteem [6], attentive behaviour [7], and increased prosocial behaviour [8,9,10]. Activities that explicitly involve interpersonal synchrony are associated with feelings of affiliation, social bonding, and a lessening of self-other distinction [11,12,13]. Decreased interpersonal synchrony is associated with moments of disagreement [14] and decreased ability to synchronize as seen in a number of disorders including Schizophrenia [15], Autism Spectrum Disorder [16], and Attention Deficit Hyperactivity Disorder [17].

Interpersonal synchrony is an explicit component and core feature of music and extends beyond playing music or singing to include many related activities such as dancing, marching, rehearsing, and listening. Multiple studies have investigated the significance and dynamics of musical synchrony. Shared movement and visual cues during music coordinate interaction and improve accuracy [18,19,20]. Musical synchrony increases a sense of shared intentionality and decreases the experience of self-other distinction [21,22,23,24], and can relate to a sense of communal identity [25,26,27]. Both a form of communication and joint action, music is intrinsically interpersonal [28,29,30]. Music is known to be related to neuroplasticity throughout life [31,32,33,34]. Neuroplasticity achieved through music, then, must be driven to some extent at the interpersonal level. The development of interpersonal measures of change at the neural level following musical interactions may identify the dynamics that drive neuroplasticity.

Studies relating the brain activity of two or more individuals (a technique that is often referred to as “hyperscanning”, for reviews see [35,36,37,38,39,40,41]) have found that the temporal dynamics of neural activity between people are more related during an interaction, a phenomenon called interpersonal neural synchrony (INS). Increased INS predicts dynamics of interaction [42], has been observed during face-to-face interactions [43,44,45] and is strongly associated with affiliation, cooperation, prosociality, and shared intention [46,47,48,49,50,51,52,53]. INS levels have also been found to predict cooperative success [54,55]. INS is increased during interactions between romantic partners [56], and induced by interpersonal touch [57]. Further highlighting connections between INS and relationships, nasal administration of oxytocin—a hormone associated with feelings of interpersonal connection—increased INS during interaction [58].

A significant number of INS studies have focused on music [38]. Due to its sustained and hierarchical temporal structure, emotional salience, and sustained synchrony, musical interaction has been posited as an ideal paradigm for interpersonal neuroscience [59]. Increased INS has been found between dyads singing and humming together, even when they were unable to see each other [60]. Increased INS between a violinist and audience members predicts the level of audience appreciation [61]. Suggesting a causal relationship, direct manipulation of INS using simultaneous, synchronized transcranial Alternating Current Stimulation (tACS) showed increased levels of behavioral synchrony in teacher/learner dyads and led to improved song learning outcomes [62].

EEG is particularly suited to music studies of interbrain synchrony due to its high temporal resolution, affording not only the ability to measure oscillatory activity at fine timescales but also precisely time-locked responses to musical events [59]. EEG studies investigating spectral changes in relation to musical events found that intrabrain alpha desynchronization musicians occur in parallel at points of reported increased emotion during performance [63,64] and that intrabrain event-related desynchronization (ERD) occurs in parallel between musicians when they intentionally coordinate with each other [65]. A series of EEG studies investigated interbrain coherence in dyads playing guitar, finding increased coherence in delta and theta band activity between participants [66,67,68]. This work, all conducted by the same team, posits that low-frequency interbrain coherence represents or is part of a mechanism that enables interpersonal temporal coordination [68]. These findings have also been replicated and extended by other researchers investigating interbrain delta and theta coherence in other types of interactions [46,69,70].

Such studies are corroborated by work that suggests brain activity in delta and theta band comprises an interface between exogenously driven and endogenously generated oscillations. A seminal study in this area showed that the rhythm of entrained oscillations is under endogenous attentional control in macaques, and can be switched to follow a specific rhythm in a set of competing rhythms [71]. In a human analogue, perceptual accuracy increases and auditory processing is more efficient when people attend to sounds that occur in a regular delta pattern, relative to an irregular one [72]. Theta-band oscillations in the auditory cortex can entrain the syllabic rate of speech [73,74,75] and help distinguish speech in background noise [76]. While more recent work in individuals has revealed a top-down dynamic to entrainment, in which persistent oscillations are sustained only at beta or gamma frequency bands, potentially driving the observed changes into lower frequency bands [77,78], interpersonal neural coherence during interaction has only been observed in lower frequencies.

Further highlighting the relevance of music and low-frequency activity is the fact that the hierarchical structure of music and brain rhythm is quite similar [79]. Both emphasize the delta band (0.5–4 Hz/30–240 bpm), as spontaneous delta band activity occurs at higher amplitudes than other frequency bands in the brain, and the beat—a key structure to which people synchronize in music—occurs almost entirely in the delta band.

Behavioral synchrony in music and rhythmic activities has been shown to continue briefly after the loss of sensory contact between people and it has been proposed that this phenomenon supports human cooperation or joint action which naturally features brief and intermittent loss of sensory contact [80]. Whether INS also persists beyond the interaction itself has remained unknown. INS beyond the period of interaction would support the idea that INS is perhaps an integral part of the mechanism by which people achieve, maintain, or recover behavioral synchrony and so represents more than parallel processing of stimuli or parallel motor activity.

The present study aimed to address this question by testing whether INS would be increased in spontaneous EEG recorded in silence after a musical interaction. A pre-post paradigm was developed in which participants grouped in dyad pairs, played a musical tapping game. Spontaneous EEG was synchronously recorded in silent, non-interactive periods immediately preceding and immediately following the musical interaction. Through comparison of identical silent, non-interactive periods that only differ by whether they precede or follow a musical interaction, we intended to avoid much of the confound of induced synchrony from simultaneous stimulation and activity.

We hypothesized that INS would be increased in the post-condition. We further hypothesized, given previous findings, that persistent effects would be found in the delta or theta band as increased wavelet power, interbrain phase coherence, or both.

## 2. Materials and Methods

### 2.1. Participants and Setup

**Figure 1:**
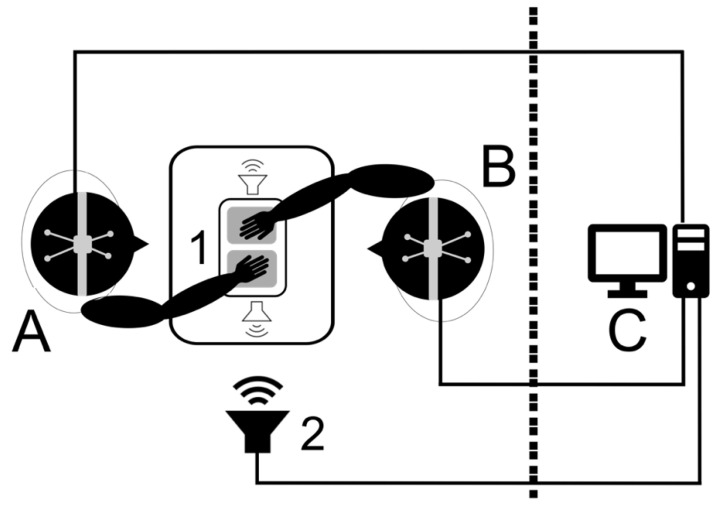
An illustration of the experimental setup during the tapping game. Participants wearing EEG caps (**A**,**B**) faced each other during the tapping game, tapping on a synth drum pad (1), and turned 90 degrees to their left during pre/post silent periods. Guide tones were played through a speaker (2) from a computer (**C**) in an adjacent room, which was also used for data collection.

Ten (10) participants were recruited through word of mouth at the department of Audiology at the University of the Pacific. Six graduate students, three faculty, and one child participated, forming ten (10) dyads, each participant undergoing the experiment twice, each time with a different, randomly-selected partner. All participants had normal hearing thresholds (>20 dB for 0.5, 1, 2, and 4 kHz) and no history of neurological disorders. Participants were between 8 and 50 years old with 3 males and 7 females. Participants were familiar with each other to varying degrees and while familiarity is known to modulate INS [81], we did not consider this to be problematic when solely establishing an effect in a pre/post paradigm. Further, not all participants were naïve to the purpose of the study. It would have been difficult for this knowledge to affect the brain-based pre/post paradigm. Participants had highly varied and diverse musical backgrounds and levels of experience. Figure 1 shows the equipment setup and participant placement. Both individuals in the dyad were seated in a sound isolated chamber facing each other. Between them, at lap height, was placed an Alesis SamplePad 4 MIDI drum pad, connected to a JBL 308P 8” studio monitor under a table directly beneath it. The drum pad was positioned so that each participant could easily reach it and tap on it by leaning forward and partly extending an arm. Participants were asked to tap with their dominant hand (all participants were right-handed). Another JBL studio monitor, connected to a computer, was placed 3′ away from the participants on a 4′ stand (Figure 1).

**Figure 2:**
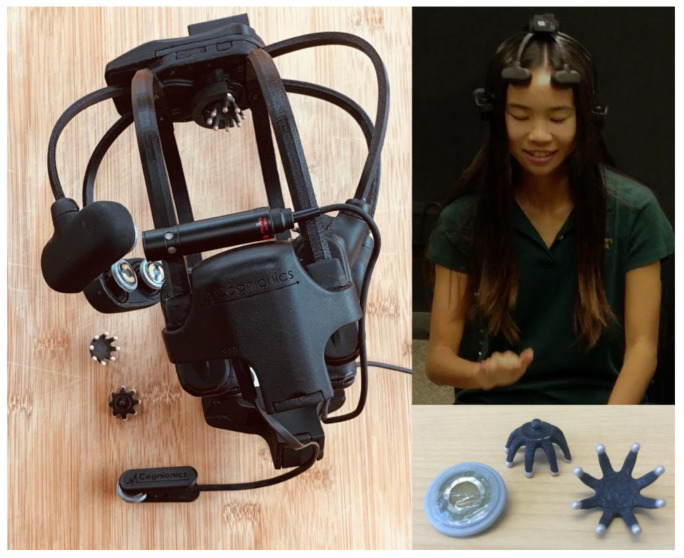
Left: Cognionics headset used for the present study (viewed from the left); right top: a research assistant tests the headset and drum machine; right bottom: tined and smooth electrodes for placement in hair and on the skin, respectively.

Each participant wore a low density, 4000 Hz sampling rate dry electrode headset made by Cognionics [82]. The design used for the present study featured 5 electrode placements: Fp1, Fp2, Cz, O1, and O2 with a forehead ground and left mastoid reference. As these headsets are not adjustable, the locations of all sites are approximate: head circumference and height will affect specific locations on each participant. Although all participant head circumferences fell within the manufacturer specified range of 52–62 cm, a custom, specially-made smaller version of the headset was used for participants with smaller heads, to align at the vertex. Two types of electrodes were used: smooth and tined. The smooth electrodes feature a soft, pliable plastic skin and are made for sitting against the bare skin of the forehead. The tined electrodes feature flexible plastic tines arranged in a star-like configuration that comb through hair to reach the scalp (Figure 2). These dry electrodes do not need any gel or any skin preparation other than swabbing bare skin with alcohol swabs. These headsets also feature two analog inputs that are recorded in sync with an EEG. These analog channels allow synchronization of EEG data with external stimuli and thus enable synchronization of recordings between headsets. For the present study, square pulses were sent from the experimental computer to both headsets. Aligning these square pulse “triggers” in the data allowed data from different headsets to be synchronized to within one sample (0.25 ms).

### 2.2. Task

The present study employs a cooperative musical activity: an anti-phase tapping game. The object of the game is to maintain anti-phase synchrony while tapping at progressively faster tempi. Anti-phase synchrony was chosen over in-phase synchrony for this study for several reasons. Trying to maintain anti-phase synchrony is a more cooperative and musical activity than in-phase, or unison, synchrony. Whether participants succeed at achieving and maintaining it, anti-phase tapping requires them to hold in mind the composite sound and focus on it rather than only their own tapping. Tapping in anti-phase synchrony has also been found to involve more significant changes in brain dynamics compared with in-phase synchronized tapping, placing significantly more demands on motor coordination than in-phase tapping [83,84,85]. Further, tapping in anti-phase synchrony also closely resembles the type of interpersonal attention and exchange required when playing music collaboratively, yet is simple and does not require pre-existing knowledge of specific musical material or performance techniques. Participants were not expected to successfully maintain anti-phase synchrony throughout their trials. Rather, attempting to do so in a game-like context was intended to be an engaging and musical form of joint action.

**Figure 3:**
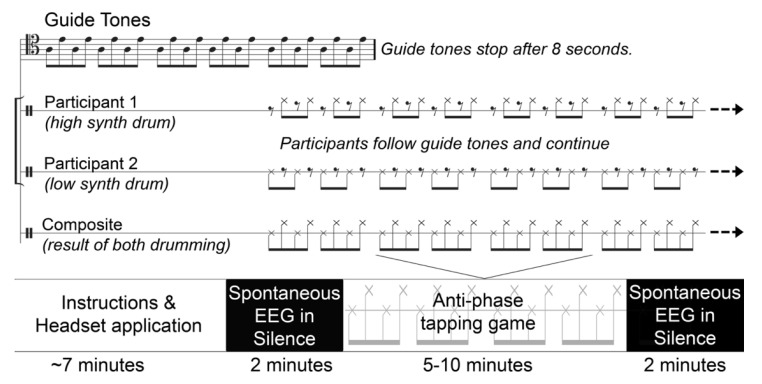
(**Top**) Representation of one instance of the anti-phase tapping game. The sequence above was repeated multiple times at increasing rates (tempi) selected by participants. (**Bottom**) Representation of the entire study procedure each dyad underwent.

An induction sequence of alternating “high” (329.6 Hz) and “low” (220 Hz) guide tones, corresponding to the musical notes “E4” and “A3”, respectively, were played through a speaker connected to a computer controlled by a researcher outside the booth. Participants were assigned at random to tap with either the higher or lower guide tone and each participant’s MIDI drum pad triggered a high or low drum sound (Garritan Timpani KS patch) corresponding to their assignment of a higher or lower guide tone. The guide tones were played for 8 s and participants were instructed to tap with their target pitch and then to maintain the composite interlocking rhythm for as long as possible after the guide tones had stopped, constituting a simple, collaborative musical game (Figure 3). In order to maximally challenge each dyad, the guide tone tempo increased across trials, and participants were instructed to move up to the most challenging pace they could perform in a sustainable manner. The initial tempo had an Interstimulus Interval (ISI) of 800 ms, decreasing 100 ms each trial until an ISI of 200 ms was reached and then further decreasing by 25 ms to 175 ms and 150 ms. Since each participant was tapping to every other stimulus, the fastest any participant was required to tap was at an interval of 300 ms. In the case that a level was so easy as to be indefinitely sustainable, participants had the option to stop when they wished and move on to the next, more challenging one. Participants were not required to complete all 9 levels but rather, were instructed to collaboratively “find the most challenging, yet sustainable tempo.” They were instructed that they were free to repeat levels they enjoyed or wanted to practice. When the dyad reached an unsustainable tempo, the game would be considered complete. The time each dyad spent tapping varied from 4.6 to 9.9 min with a mean of 6.43 and standard deviation of 1.93 min.

### 2.3. Procedure

Participants received instructions while EEG headsets were applied and impedances checked. All impedances were brought below 300 kΩ and signal quality was visually checked and monitored throughout the recordings. The instructions did not include practice trials since doing so could possibly induce the brain dynamics that the post-task EEG was intended to measure. Rather, participants received verbal instructions and the first few trials were set to a pace that would facilitate learning and familiarity with the task. The EEG recording was signaled with a chime: participants would sit in silence, turn 90 degrees to their left (leaning slightly forward so that they could not see each other) for a period of 2 min. Participants were observed through a window to verify that they were in compliance with these conditions. When this was complete, a second chime would cue them to face each other and begin the task (described above). Following this, the participants would begin the game. When the game was complete, signaled by participants by again turning 90 degrees to their left, such that they could not see each other, a second EEG recording would take place as they sat in silence with their eyes open for 2 min. A final chime would indicate that the study was complete (Figure 3). While an EEG was recorded throughout the game, here we focus on the spontaneous EEG prior to and following the tapping game, to examine if any changes are present between these two conditions.

## 3. Data Analysis

### 3.1. EEG Processing

EEG analysis included three pre-processing steps: (1) temporal alignment of the two EEG files from a participant dyad using recorded triggers, (2) data filtering to remove low-frequency drift and dc offset and emphasize cortical activity, and (3) selection of artifact-free sections of data in the pre- and post-activity sections from each participant. To align the dyads’ files in real-time, time zero was adjusted to the initial trigger (T_0_) that was simultaneously sent to each recording at the beginning of each experiment. Continuous data from both participants in a dyad were overlaid to verify that T_0_ and subsequent triggers were aligned across the entire recording session. Data files were then filtered with a Finite Impulse Response (FIR) band-pass from 0.5 to 60 Hz. Finally, overlapping sections of EEG data free from eyeblink and EMG (i.e., clean in both participants at the same time in the recording) were identified and the first 15 s doubly-clean (artifact-free in both participants) segment was selected for analysis from pre- (PRE) and post-activity (POST) sections. The 15 s duration was selected in order to systematize section durations and maximize available data for spectral analysis as concomitantly clean durations of EEG beyond 15 s were not regularly observed across dyad data and several dyads did not reach 15 s. This resulted in a mean duration of 14.77 s (SD = 0.48) in the PRE condition and a mean of 14.78 s (SD = 1.31) in the POST condition. Clean sections began on average about one minute into the PRE section (M = 0.97 min, SD = 0.59 min), and on average were within the first 30 s following the end of tapping in the POST section (M = 0.45 min, SD = 0.56 min). There were no significant differences between clean section duration (t_(9)_ = 0.20, *p* = 0.984) or time of occurrence (t_(9)_ = 1.983, *p* = 0.079) across conditions.

While Independent Component Analysis (ICA) would seem an effective method for cleaning data, the mobile EEG headsets used in the present study had only five channels, a number we deemed insufficient for reliable ICA results. While an obvious disadvantage for ICA, the choice to use such low-density headsets is directed towards scaling up numbers of participants in future studies.

A combination of wavelet analysis and cross-correlation was used to obtain measures of INS. Wavelet analysis, such as Fourier decomposition, expands a function in terms of scaling properties that are localized in both time and frequency [86]. Here, we used a Morlet wavelet function [87], similar to a modulated Gaussian waveform, with a mother wavelet size of six cycles (ῳ_0_ = 6) in frequency bands between 1 and 100 Hz. Power and instantaneous phase were then extracted by taking the absolute and angle values of the wavelet output for each time point and frequency.

Wavelet power was averaged within two frequency bands: delta (defined here as 0.5–3 Hz to avoid overlap) and theta (4–8 Hz). To determine the degree of INS in a dyad, we cross-correlated the instantaneous phase vector for each frequency bin within delta and theta bands. For each frequency bin, the maximum cross-correlation *r* within a window of −5 and 5 ms lag around 0 ms was extracted in order to assess nearly-simultaneous inter-brain phase correlation, rather than inter-brain phase correlation that was offset by some time factor. By taking the maximum *r*-value over this time period, we also avoided wrap-around phase effects and antiphase correlations. While one might expect antiphase rhythms would be induced by an antiphase game, we focus here on the composite rhythm as attention to it is key to a sustained interaction. The maximum correlation across the frequency bins within each band was then selected. The result of this computation was a matrix of values that described how well the phase of neuronal oscillations in the delta and theta frequencies correlated between dyad participants in PRE and POST conditions.

### 3.2. Statistical Analysis

The two dependent variables in this dataset were phase correlation (r) and power (pwr). The predictor was condition (PRE, POST) and data were collected from a sample of ten dyads. Brain response measurements were taken from five channel locations (Fp1, Fp2, Cz, O1, O2) in two frequency bands (delta, theta). In the case of phase correlation, we have one measurement for each dyad (i.e., the correlation of phase between the two subjects), giving 200 observations and a balanced design. We used a linear mixed-effects model to determine statistical effects. Dyad was specified as the subject variable. Condition, channel, and frequency band were specified as repeated variables. The subject variable was modeled as a random effect and the repeated measures variables were modeled as fixed effects.

For power, we had two measurements per dyad (i.e., the power for each subject) and the same repeated variables, giving 400 observations and a balanced design. We again used a linear mixed-effects model to determine effects. Dyad and subject were specified as the subject variables. As in the phase correlation analysis, condition, channel, and frequency band were specified as repeated variables. Again, the subject variables were modeled random effects and the repeated measures variables were modeled as fixed effects.

In both analyses, Type III tests showed which fixed effects were related to the dependent variable of brain response measurements. Post-hoc paired sample t-tests were conducted to clarify differences when significant main effects of condition (i.e., PRE vs. POST) were observed.

The total duration of tapping for each dyad across all trials was in addition correlated with power magnitude and phase correlation values in delta and theta bands to determine if the strength of INS varied with the length of time playing together.

## 4. Results

### 4.1. Wavelet Power

**Figure 4:**
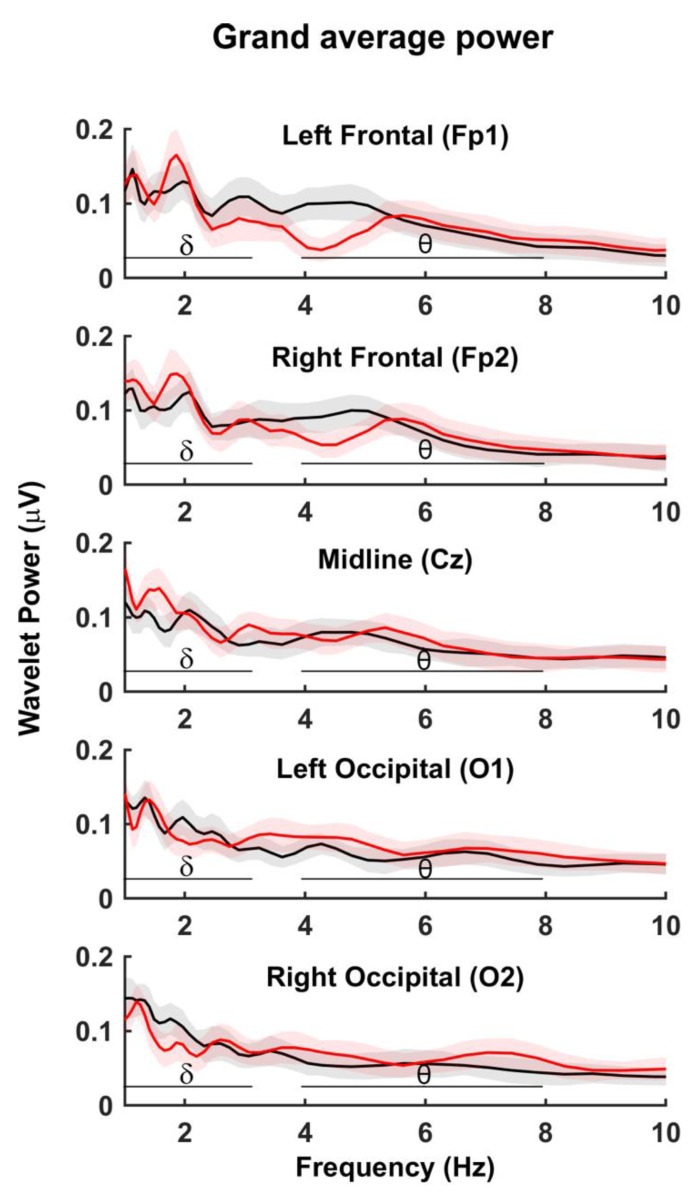
Grand average wavelet power at each of the 5 electrode sites. PRE (black) POST (red). Shading shows standard error and horizontal bars show delta and theta band analysis regions.

To determine whether power differed in the PRE vs. POST condition, a mixed-effects model was conducted with one dependent variable (power), 20 levels of repeated effects (two conditions*five channels*two frequency bands), and 12 levels of random effects (two subjects + ten dyads). Results showed significant main effects for the channel (F_(4/70.2)_ = 28.188, *p* < 0.001) and frequency band (F_(1/138.59)_ = 57.22, *p* < 0.001), but no effect of condition (F_(1/138.59)_ = 0.820, *p* = 0.367) and no interaction between the three independent variables (F_(4/138.59)_ = 0.432, *p* = 0.785). Examination of the data showed the expected topographical differences in the distribution of power across the scalp, which was greatest in frontal electrodes. In sum, wavelet power did not significantly change from PRE to POST conditions, nor were there any channel-specific interactions with the condition. Figure 4 shows the grand average wavelet power across frequency for all channels. Delta (δ) and theta (θ) frequency ranges are shown with horizontal lines. Frontal channels do appear to have a significant reduction in PRE to POST power around 4Hz at the lower theta range.

### 4.2. Interpersonal Neural Synchrony—Frequency Specific Phase Coupling

INS was measured as the maximal inter-individual phase correlation within the delta and theta bands. To determine whether phase correlation differed in the PRE vs. POST condition, a mixed effects model was conducted with one dependent variable (r), 20 levels of repeated effects (two conditions*five channels*two frequency bands) and 10 levels of random effects (10 dyads). Results showed significant main effects for channel (F_(4/59.28)_ = 10.906, *p* < 0.001), frequency band (F_(1/107.77)_ = 297.347, *p* < 0.001) and condition (F_(1/107.77)_ = 8.497, *p* = 0.004) as well as interaction between the three independent variables (F_(4/59.28)_ = 3.358, *p* = 0.015).

**Figure 5:**
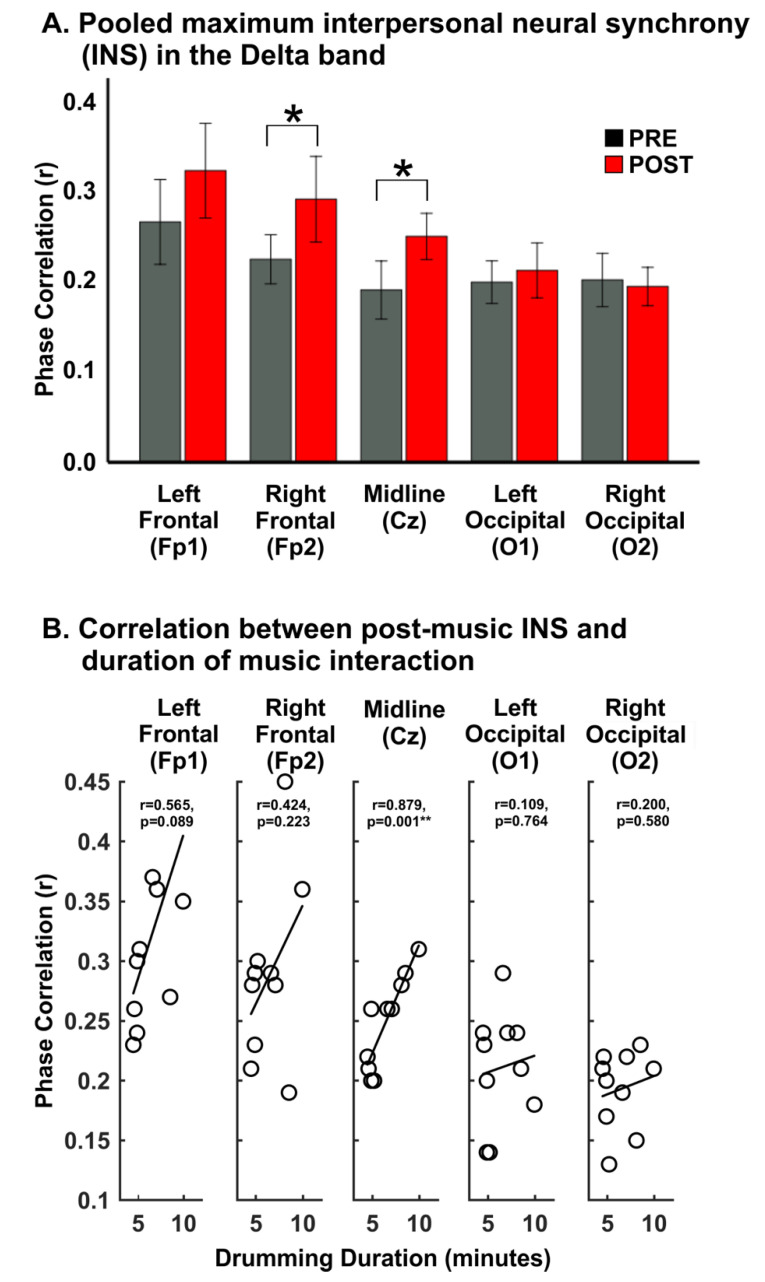
**(A**) Bar graphs representing mean phase correlation in PRE (grey) and POST (red) conditions at each of the five electrode sites used for the study. Statistical significance is indicated by the asterisks (*). (**B**) Correlations between duration of musical interaction and POST condition delta band INS.

Post-hoc, paired samples t-test analysis showed that phase correlation in the delta band was greater in the POST condition, compared to PRE, at midline (Cz) (t_(9)_ = 3.130, *p* = 0.012) and right frontal (Fp2) (t_(9)_ = 2.289, *p* = 0.048) channels. Taken together, this means that delta frequency INS is greater between dyad participants after musical interaction, and especially at electrodes over the central and right frontal brain areas. Figure 5A shows the mean and standard error of the phase correlation values in the delta band, illustrating the findings. No effect of condition (i.e., PRE vs. POST) was observed in the theta band data, suggesting that theta-band INS is similar before and after musical interaction.

### 4.3. Relationships between Interpersonal Neural Synchrony and Duration of Musical Interaction

To determine putative brain-behavior relationships, Pearson’s correlations were computed between the duration of musical interaction and delta-mediated INS in both the PRE and POST conditions. Results (Figure 5B) showed a significant correlation between delta-mediated INS and tapping duration at Cz (r = 0.879, *p* = 0.001) only for the POST condition, such that longer tapping duration was associated with greater post-tapping INS, but did not relate to pre-tapping INS. There was a similar trend for frontal electrodes though it did not reach significance. This result suggests a positive relationship between the behavior of musical interaction and the degree of interpersonal brain coherence after the behavior. The lack of significant behavioral correlations with PRE-tapping INS suggests that pre-interaction INS does not relate to the duration of the interaction, and argues against the POST finding being due to simple systematic bias. No significant correlations were observed between theta-band INS and musical interaction duration.

### 4.4. Tapping Characterization

**Figure 6:**
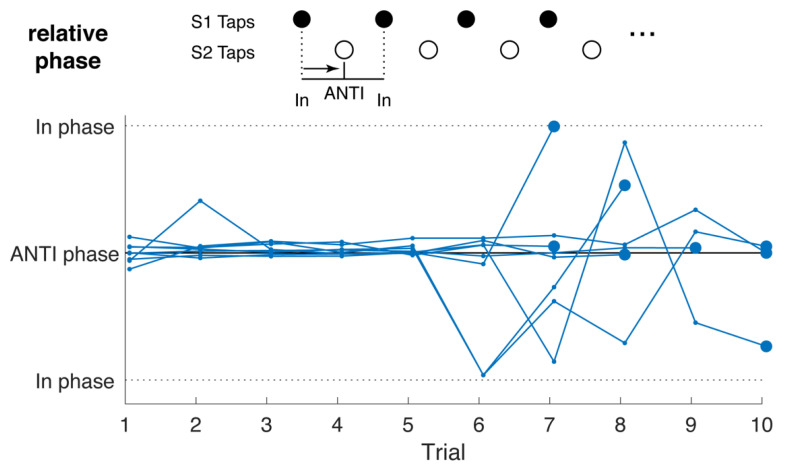
Relative phase per trial for participant dyads. Each dyad’s trials comprise blue dots connected by a blue line. The final trial of each dyad is marked in bold.

Each participant’s taps were recorded by three-axis accelerometers and analog audio channels on each headset. The high level of freedom in the musical interaction resulted in significant variability in terms of the number of trials, tempi achieved, and stability of anti-phase synchrony. Relative phase calculations for each dyad show that all dyads were able to maintain anti-phase synchrony as the tempo increased across trials, with relative phase variability increasing in some dyads towards the final trials, which featured higher tempi (Figure 6). From this, it can be inferred that in no case were PRE/POST changes in INS the result of anti-phase tapping collapsing to in-phase tapping, a phenomenon that could be expected to occur frequently when tempi become challenging [88].

## 5. Discussion

This study explored the possibility of persistent oscillatory changes in interpersonal brain activity following a dyadic musical interaction. The main finding, a significant increase in delta band INS after a musical interaction, is novel to the authors’ knowledge and holds significant implications for the investigation of music-related neuroplasticity at the interindividual level. Further, that the effect was seen only in the Delta band may highlight the importance of the musical beat, which corresponds closely to this frequency band in practically all cultures [89]. This result complements the work of Nguyen et al., which found that the synchrony of cortical hemodynamic activation between mother-child subject pairs appeared to continue into rest periods between cooperative interaction [54]. While this could be interpreted as a post-interaction continuation of INS, the study design did not include a pre-task resting period and so the degree to which INS during between-task resting periods may differ from baseline mother-child INS was left unknown [54]. This result also complements the work of Gugnowska et al., who found amplitude-based INS increased in gamma band between two pianists during pauses in play [90]. Future studies will be needed to investigate the relationship between the temporal dynamics of these amplitude changes and the lower frequency phase coupling investigated in the present paper. The findings of the present study also complement past work, which focused on findings of increased INS during an interaction. A persistent issue with INS measured during interaction is that it may simply result because individuals in interaction are receiving simultaneous multisensory input and also engaging motor pathways in time with the simultaneous behavioral interaction [91,92,93,94]. While it is difficult to disambiguate more mundane or trivial forms of INS during interaction in the form of parallel processing from potential mechanisms of maintaining synchrony, the present finding of increased INS during a POST interaction period suggests the latter. We provide some preliminary interpretations of this surprising finding below, focusing on neuroplasticity behavioral significance, and neural dynamics, with suggestions for follow-on research to more fully understand this post-interaction INS phenomenon.

Li et al. [51] found that basketball players—whose sport requires very close synchrony between players—achieved INS more quickly and at higher levels than age-matched controls during a cooperative drawing task, which they also completed more quickly than controls. This suggests a long-term cumulative effect stemming from neuroplasticity: people who frequently engage in joint action, such as music or team sports, may adapt for those activities at the neural level, possibly becoming more efficient at them. In the present study, INS was found increased relating to the length of time each dyad played together, suggesting that there may also be a more immediate cumulative effect. At the same time, it is possible that dyads that performed well, or perhaps were just more comfortable with the game due to greater capacity to synchronize, naturally played for longer durations, accounting for this effect. In future studies, we intend to investigate these issues as part of an ongoing longitudinal study, facilitating tracking effect size with the magnitude of observed INS.

Koban et al. [95] propose that behavioral and neural synchrony can be explained from the perspective of optimization theory: being in synchrony allows conservation of computational resources and also causes alignment—and overlap—in self-other representations. Music, then, could facilitate further optimization as it comprises a temporal filter for behavior, organizing interaction into easily predictable rhythms. In line with this, we propose a corollary that it may also be more efficient to maintain these rhythms after an interaction in order, for example, to restart the interaction more easily. Supporting this, many interactions do not have well-defined endings. For example, a recent study found that 70% of adults were not able to determine when a conversation had ended [96]. Therefore a potential benefit exists for maintaining some kind of latent representation of the interaction dynamics (e.g., the rhythms of INS) until some new stimulus or situation occurs. Our findings would be consistent with this idea.

Beyond optimizing energetic and computational resources while maintaining a sense of affiliation, there would be a strong goal-oriented benefit in maintaining the rhythms of interaction. Many group behaviors from hunting to basketball feature moments in which perceptual contact between group members is lost [80]. Maintaining the group rhythm (a form of covert synchrony) across these moments would be crucial for success. This phenomenon has been observed at the behavioral level [80]. We propose that the persistent neural synchrony observed in the present study may represent a neural correlate, an interbrain dynamic that is related to this ability.

Perhaps the most well-known—and highly-debated—potential mechanism related to our findings would be neural entrainment. Sustained phasic oscillations persisting well after periodic stimulation has ceased would be compelling evidence for the classic entrainment models posited by theories such as the Dynamic Attending Theory (DAT) [97]. However, even at the individual level, the persistence of endogenous oscillations phase-locked to a low-frequency periodic stimulus has so far been reported for only a few cycles [98,99,100]. In this light, the present finding of what could be interpreted as evidence of entrained low-frequency rhythm persisting many seconds after an interaction between two brains is startling indeed. That said, some studies have found direct evidence for phase-locked entrainment at higher frequencies [77,101], supporting an alternative “top-down” phase reset model which could help account for such a finding [78].

Further complicating simple entrainment by phase reset interpretation would be that, as described above, virtually every trial of the anti-phase tapping game ended in the collapse of the anti-phase rhythm the participants were attempting to maintain. In each trial, participants’ tapping would fall out of synchrony or occasionally collapse briefly into in-phase synchrony. If the observed increases in INS represent continued synchronous oscillations resulting from a phase reset process, then the observed INS would more likely be related not to the periods of anti-phase tapping synchrony that invariably occurred earlier in each trial but rather to the chaotic or in-phase tapping synchrony that occurred at the end of each trial. The question of whether anti-phase tapping synchrony or in-phase tapping synchrony would be causally related to the observed effect can only be addressed through future studies that directly compare pre/post interpersonal phase coherence between anti-phase and in-phase tapping. Further, now that the existence of increased post-interaction INS has been established in a musical context, pre/post INS must be compared between tapping and control activities featuring other forms of interaction, such as conversation.

An alternative possibility to passive entrainment caused solely by phase reset of neural firing patterns at low frequencies would be an active attention/cooperation-driven form of entrainment. Participants might covertly or subconsciously replay their experience. This would achieve all the behavioral level benefits described above: optimization of energetic and computational resources, the scaffolding of potential continuance of interaction, and be evident seconds after discontinuation of activity. Such “covert replay” could result in the observed effect. Indeed, covert motor simulation has been posited repeatedly as an important behavioral and perceptual scaffold (reviewed in [102,103]) and it is known that internal rehearsal or imagery can impact brain oscillations [104]. Further supporting this idea, INS has been linked to explicit action plans during pauses in joint activity [90].

These ideas are eminently testable and invite future research. For example, the hypothesis that post-interaction INS relates to some advantage for restarting the interaction could be directly tested by examining how effectively interaction behaviors restart after a POST break. We would predict that dyads with higher POST INS would more effectively re-start the interaction. It would also be possible, using a simple interview format, to determine which participants experienced or engaged in mental imagery or active rehearsal of dynamics during the break. We would predict that dyads who were aware of such processes would more effectively re-start the interaction.

The important brain-behavior finding, that duration of interaction was significantly correlated with INS, could index the inherent ability of one or both of the participants. Better synchronizers would likely have a larger effect and be able to synchronize for more sustained periods. A very good synchronizer might also be able to adapt rhythmically to a less able one and so may still be able to carry the interaction for long periods, possibly also driving INS. We plan in future studies to characterize or compare the musical interaction (e.g., Did participants feel successful? Were they able to maintain synchrony at a high level before giving up? Did they enjoy the interaction?) and how such characterizations relate to observed effect sizes.

The present study did not compare brain dynamics during interaction with pre- or post- conditions. This is because during interaction overt movement (and possible movement artifacts) are synchronized, potentially inflating the measured INS and making the comparison difficult. In future work, we plan to develop methods that would facilitate such comparison. It may be that the antiphase condition will mitigate this to some extent since behavior will be out of phase, although the dyad still receives the same auditory inputs.

While the participants were somewhat familiar with each other, and it is known that familiarity modulates INS [81], this familiarity cannot explain the observed effect in the POST condition: the key variable between PRE and POST was the tapping game. However, familiarity and prosociality will need to be investigated in future work in this context.

In closing, we wish to emphasize that the observed post-interaction changes in brain dynamics only become apparent by taking the dyad as the fundamental unit of analysis. It may be that post-interaction EEG changes in stereotypical ways, making it nearly impossible to detect sustained changes in a single brain. While further study will clearly be needed to investigate this phenomenon, it seems apparent that an advantageous and perhaps necessary approach would involve multi-person EEG recording. It may, in fact, take two.

## Data Availability

Data used for this study can be found in Appendix B in Table A2 and Table A3.

## Appendix A

**Table A1:** Marginal Means and Standard Errors of Wavelet Power in PRE and POST conditions.

Condition	Channels	Delta Power (SE)	Theta Power (SE)
PRE (before rhythm activity)	Fp1	14.3 (1.9)	7.1 (1.0)
	Fp2	13.3 (1.9)	6.9 (1.0)
	Cz	5.7 (0.7)	4.5 (0.5)
	O1	7.9 (2.2)	4.9 (0.8)
	O2	10.4 (3.4)	5.3 (0.8)
POST (after rhythm activity)	Fp1	17.6 (2.5)	7.0 (0.9)
	Fp2	17.4 (2.4)	6.9 (0.9)
	Cz	6.9 (0.9)	4.5 (0.5)
	O1	8.5 (3.5)	4.6 (1.1)
	O2	9.0 (2.4)	4.6 (0.8)

## Appendix B

Individual Dyad Inter-Brain Correlations Underlying Figure 4.

**Table A2:** Dyad INS in delta band.

Maximum Inter-Brain Correlation, Delta Band							
Condition Dyad			R Deltamax Fp1	R Deltamax Fp2	R Deltamax Cz	R Deltamax O1	R Deltamax O2
PRE	1		0.19	0.17	0.19	0.13	0.15
	2		0.24	0.25	0.16	0.23	0.18
	3		0.22	0.21	0.15	0.20	0.27
	4		0.20	0.18	0.27	0.25	0.16
	5		0.44	0.27	0.10	0.16	0.20
	6		0.23	0.15	0.17	0.18	0.17
	7		0.30	0.28	0.19	0.17	0.23
	8		0.28	0.24	0.26	0.23	0.26
	9		0.22	0.23	0.20	0.19	0.23
	10		0.30	0.24	0.21	0.23	0.13
		Mean	0.2634	0.2225	0.1888	0.1974	0.1998
		Std. Error of Mean	0.02327	0.01348	0.01596	0.01169	0.01460
POST	1		0.37	0.29	0.26	0.29	0.19
	2		0.51	0.45	0.28	0.24	0.15
	3		0.26	0.28	0.21	0.23	0.22
	4		0.31	0.30	0.17	0.14	0.13
	5		0.23	0.21	0.22	0.24	0.21
	6		0.36	0.28	0.26	0.24	0.22
	7		0.27	0.19	0.29	0.21	0.23
	8		0.35	0.36	0.31	0.18	0.21
	9		0.30	0.29	0.26	0.20	0.17
	10		0.24	0.23	0.20	0.14	0.20
		Mean	0.3197	0.2883	0.2446	0.2103	0.1927
		Std. Error of Mean	0.02595	0.02352	0.01426	0.01510	0.01050

**Table A3:** Dyad INS summaries data for theta band.

Maximum Inter-Brain Correlation, Theta Band							
Condition Dyad			R Thetamax Fp1	R Thetamax Fp2	R Thetamax Cz	R Thetamax O1	R Thetamax O2
PRE	1		0.16	0.14	0.15	0.11	0.08
	2		0.16	0.13	0.11	0.08	0.08
	3		0.19	0.17	0.14	0.09	0.08
	4		0.20	0.20	0.16	0.12	0.12
	5		0.14	0.10	0.11	0.09	0.05
	6		0.10	0.12	0.12	0.06	0.07
	7		0.14	0.08	0.13	0.10	0.08
	8		0.10	0.11	0.09	0.15	0.15
	9		0.15	0.17	0.14	0.10	0.10
	10		0.13	0.12	0.09	0.08	0.14
		Mean	0.1467	0.1349	0.1256	0.0985	0.0955
		Std. Error of Mean	0.01044	0.01150	0.00759	0.00775	0.01020
POST	1		0.16	0.13	0.13	0.10	0.07
	2		0.15	0.19	0.12	0.07	0.09
	3		0.15	0.12	0.16	0.18	0.10
	4		0.06	0.08	0.09	0.11	0.12
	5		0.14	0.16	0.19	0.12	0.14
	6		0.10	0.09	0.08	0.09	0.18
	7		0.13	0.08	0.10	0.12	0.10
	8		0.06	0.07	0.13	0.15	0.08
	9		0.17	0.18	0.14	0.15	0.17
	10		0.13	0.11	0.11	0.09	0.13
		Mean	0.1256	0.1208	0.1245	0.1187	0.1171
		Std. Error of Mean	0.01205	0.01362	0.01038	0.01040	0.01211

**Table A4:** Dyad-specific duration of the interaction.

Dyad	Duration (Seconds)
1	594
2	534
3	486
4	378
5	366
6	318
7	312
8	300
9	294
10	276
